# Hemodynamic variations in arterial wave reflection associated with the application of increasing levels of PEEP in healthy subjects

**DOI:** 10.1038/s41598-022-07410-1

**Published:** 2022-02-28

**Authors:** Jacopo Belfiore, Etrusca Brogi, Niccolo Nicolini, Davide Deffenu, Francesco Forfori, Carlo Palombo

**Affiliations:** Division of Anaesthesia and Intensive Care, Department of Surgical, Medical, Molecular and Critical Care Area, University of Pisa, Via Paradisa 2, 56124 Pisa, Italy

**Keywords:** Respiration, Heart failure

## Abstract

Positive end-expiratory pressure (PEEP) may affect arterial wave propagation and reflection, thus influencing ventricular loading conditions. The aim of the study was to investigate the hemodynamic variations in arterial wave reflection (i.e., wave reflection time, augmentation index, left ventricular ejection time, diastolic time, SEVR) associated with the application of increasing levels of PEEP in healthy subjects. We conducted a prospective observational study. Study population was selected from students and staff. Pulse contour wave analysis was performed from the right carotid artery during stepwise increase in PEEP levels (from 0 cmH_2_O, 5 cmH_2_0, 10 cmH_2_O) with applanation tonometry. Sixty-two healthy volunteers were recruited. There were no significant changes in heart rate, augmentation index (AIx), left ventricular ejection time, Diastolic time (DT) among all of the different steps. A significant increase of time to the inflection point (Ti) was observed during all steps of the study. Diastolic area under the curve (AUC) divided by systolic-AUC (SEVR) increased from baseline to PEEP = 5 cmH_2_O, and from baseline to PEEP = 10 cmH_2_O. AIx and Ti were significantly correlated (directly) at the baseline and during PEEP = 10 cmH_2_O. Ti and DT were significantly correlated at the baseline and during PEEP = 5 cmH_2_O. In our preliminary results, low levels of PEEP played a role in the interaction between the heart and the vascular system, apparently mediated by a prolongation of the diastolic phase and a reduction in the systolic work of the heart.

Clinical trials registration number: NCT03294928, 19/09/2017.

## Introduction

A Positive End-Expiratory Pressure (PEEP) during mechanical ventilation is commonly applied to prevent alveolar collapse, recruit and stabilize lung units, improve oxygenation, increase functional residual capacity and lung compliance^1,2^. Furthermore, PEEP may help the respiratory muscles to decrease the work of breathing in some conditions.

In light of these positive effects, PEEP is used in various clinical scenarios including pulmonary oedema (where it promotes redistribution of extravascular lung water and improves oxygenation and lung compliance)^3^ and acute respiratory distress syndrome (where oxygenation can be increased by keeping alveolar units opened at the end of expiration)^4^. Low levels of PEEP increase intra-alveolar volume in pervious alveoli while higher levels of PEEP are useful for alveolar recruitment: both of them improve functional residual capacity (FRC)^2^. Finally, PEEP is applied during intraoperative ventilation and after surgery in order to prevent and treat atelectasis^5^.

However, the application of high PEEP level may also lead to adverse consequences such as incomplete expiration, air-trapping (intrinsic PEEP) and increased risk of barotrauma^6^. Apart from pulmonary effects of PEEP, hemodynamic consequences are controversial and not completely understood^7,8^. Many studies have demonstrated that alveolar recruitment manoeuvres (using high levels of PEEP) increase right ventricular afterload and decrease right ventricular preload, similarly to a Valsalva effect^9^. Moreover, augmented intrathoracic pressure induced by high level of PEEP, decreases right ventricle and increases left ventricle end—diastolic volume (EDV) as described also by transmitral pulsed-wave Doppler (PW-D)^10^. At the same time, PEEP decrease left ventricle afterload^11^. Consequently, to foresee the hemodynamic consequences of PEEP application is challenging in critically ill patients.

Arterial contour waveform analysis (CWA) is increasingly proposed as tool to estimate the hemodynamic status of a critical care patient as well as essential information about the arterial wall integrity and stiffness^12^. Hemodynamic effects of PEEP strongly depend also on arterial function and arterial properties (i.e., stiffness and peripheral tone)^13^, consequently, it will be useful to examine the haemodynamic impact of PEEP using an arterial waveform analysis. CWA can be evaluated with different non-invasive and reproducible techniques^14–16^. Among them, applanation tonometry is a non-invasive measurement capable to perform CWA on central arteries (carotid artery)^17^ that can be practically used in the intensive care unit (ICU). This technique enables the estimation of wave reflection through the analysis of blood pressure waveform.

However, in critically ill patients, several confounding factors may hinder the interpretation of the hemodynamic effects of PEEP application. Thus, the goal of our study was to investigate in young healthy subjects, with normal cardiovascular compliance and arterial tone, the direct effects of stepwise increase in PEEP. "We aimed to evaluate, in young healthy subjects, the feasibility of carotid pressure waveform analysis in order to detect changes in arterial and LV function associated with various levels of PEEP. Secondary end-point was to assess the best level of PEEP from the hemodynamic point of view, at least in young healthy subjects, in order to have a reference for evaluation of patients in a clinical setting.

## Methods

After approval of the Local Research Ethics Committee of Pisa University Hospital, a prospective observational study was conducted in a single university center in a 2 months period. Written informed consent was obtained from all the participants in the study. Procedure was carried out in accordance with the Declaration of Helsinki (2000).

### Study population

Population in study was selected from students and staff of the University of Pisa. The inclusion criteria were as follows: age between 18 and 40, absence of any chronic diseases, no active smoker, no story of drug abuse, no use of caffeine the day of the exam. We excluded patients with pregnancy, and intake of caffeine the day of the exam.

### Study design

The design of the study consisted in four-step measurements of central artery blood pressure evaluating contour wave analysis during a progressive increase of PEEP level. Subjects rested supine for 10 min before starting; recordings were performed in the morning, 2–4 h after light breakfast, abstaining from smoking and caffeinated beverages. Each step takes two minutes to be performed.

#### First step (Baseline)

Spontaneously breath in air at atmospheric pressure during applanation tonometry.

#### Second step (PEEP = 5 cm H_2_O)

Applanation tonometry performed during the application of PEEP = 5 cmH_2_O.

#### Third step (PEEP = 10 cm H_2_O)

Applanation tonometry performed during the application of PEEP = 10 cmH_2_O.

#### Fourth step (recovery)

Spontaneously rebreating air at atmospheric pressure during applanation tonometry.

Contour wave analysis was performed with applanation tonometry technique using a hand-held tonometer probe (PulsePen**®**; DiaTecne Srl, Milano, Italy^18^). The applanation tonometry technique consists of locating the sensor over the skin where the artery is found and apply a moderate pressure in order to slightly compress the artery. Thus, the sensor records the pressure in the middle of the compressed artery. A PEEP valve was connected to the “face mask-Ambu bag” system in order to set each increasing level of PEEP^19^. Subjects were breathing spontaneously.

### Blood pressure (BP) and contour arterial waveform analysis (CWA)

At the beginning of the test, arterial blood pressure (BP) was measured with a standard adult cuff on the left brachial artery, in order to calibrate waveforms using the oscillometric method^20,21^.

Right carotid arterial pulse waves were recorded with an arterial tonometer sensor array integrated with an electrocardiogram unit for a total of four times per patient (one time for each step). In the study the tonometer was applied to the right carotid artery without head support. The shape of the peripheral pulse wave was captured electronically using a laptop computer linked to a desktop (PulsePen pulse wave analysis system). The PulsePen system generates an average peripheral pulse wave contour from a 12.5 s period^18^. Tonometry has a high sensitivity to sensor position and angle.

The following parameters were obtained from pulse wave analysis^18,22^:

- Augmentation pressure (AP): the amount by which central pressure increases due to reflected wave, expressed in mmHg.
- Pulse pressure (PP): the pressure difference between the systolic and diastolic pressures, expressed in mmHg.
- Augmentation index (AIx): defined as reflected wave amplitude divided by pulse pressure, expressed as a percentage (AP/PP).
- Time to the inflection point (Ti): is measured from the foot of the forward traveling pressure wave to the foot of the reflected wave, expressed in msec.
- Left Ventricular Ejection Time (LVET): duration of left ventricular systolic ejection in msec.
- Diastolic time (DT): duration of left ventricular diastole in msec.
- SEVR: diastolic area under the curve (AUC) divided by systolic-AUC (Buckberg index)
- Heart rate (HR) in beats per minute (bpm)
- Mean systolic blood pressure (MSBP): the mean of the systolic blood pressure (mmHg)
- Mean diastolic blood pressure (MDBP) the mean of the diastolic blood pressure (mmHg)

All measurements taken on single patients were performed by the same operator (JB) and only high-quality pulse wave traces were considered acceptable. We also calculated the intra-observer reproducibility, computed as the percentage of the absolute difference between two sets of measurements, divided by their mean. Consequently, these measurements allow analysing the degree of agreement of measurements by the same observer (intra-observer) on the same patient.

After the measurements were performed, a case report form was filled out with participant ID, age, weight, height, Augmentation pressure, Pulse pressure, AIx, Ti, Left Ventricular Ejection Time, Diastolic time, SEVR, Heart rate, Mean systolic blood pressure, Mean diastolic blood pressure.

Main goal of this work was to investigate the variations in wave reflection time (Ti) and augmentation index (AIx) induced by the application of different stepwise levels of PEEP in healthy subjects. Secondary aim was to assess the effects of stepwise PEEP on cardiac indices such as left ventricular ejection time (LVET), diastolic time (DT), SEVR induced by the application of different level of PEEP.

### Statistical approach

In order to calculate the sample size, we followed the recommendations of Charan and Biswas^23^. We calculated our sample size assuming that a standard deviation (SD) in population was about 11^24^ and an effect size (d) was 5.8%^25^. The precise estimation sample size was 56; therefore, we decided to recruit a total of 62 participants in order to have sufficient statistical power.

Data were analyzed using Stata 11.1 software (Stata Corp, College Station, TX). Data were expressed as mean values ± SD. Student’s t-test was used for assessment of intragroup changes between two different steps (R-2.15.1, Free Software Foundation's GNU project). Pearson’s linear regression analysis (R-2.15.1, Free Software Foundation's GNU project) was used to investigate relationship between two parameters in the same step. Intraclass parameter modifications among all of the different steps were calculated using one-way analysis of variance (ANOVA). A *p*-value < 0.05 was considered to be significant.

### Research involving human participants and/or animals

Approval of the Local Research Ethics Committee of Pisa. Procedure was carried out in accordance with the Declaration of Helsinki (2000). All procedures performed in studies involving human participants were in accordance with the ethical standards of the institutional and/or national research committee and with the 1964 Helsinki declaration and its later amendments or comparable ethical standards”.

### Informed consent

Informed consent was obtained from all individual participants included in the study.

### RB Information

This study was approved by the Local Research Ethics Committee of Pisa (protocol number n°43004, date of approval 12/09/2017).

## Results

We recruited 62 healthy volunteers, 31 males and 31 females (aged between 20 and 35 years old); all subjects were apparently healthy, with not known cardiovascular disease or major cardiovascular risk factors, and none was receiving any medication. General characteristics of the study population were reported in Table 1. Intra-observer variability of measurements was between 2.5% for LVET and 5.7% for AIx.

**Table 1:** Demographic characteristics of the population in study.

Parameters	Mean	SD
AGE (year)	26.8	3.1
HEIGHT (cm)	172	14
WEIGHT (Kg)	68.7	7.8
BMI	20.5	2.4
HR (bpm)	75	16
SBP (mmHg)	116	12
DBP (mmHg)	77	12

Data are expressed as mean ± standard deviation (SD).

BMI, body mass index; HR, heart rate; SBP, systolic blood pressure; DBP, diastolic blood pressure.

**Figure 1:**
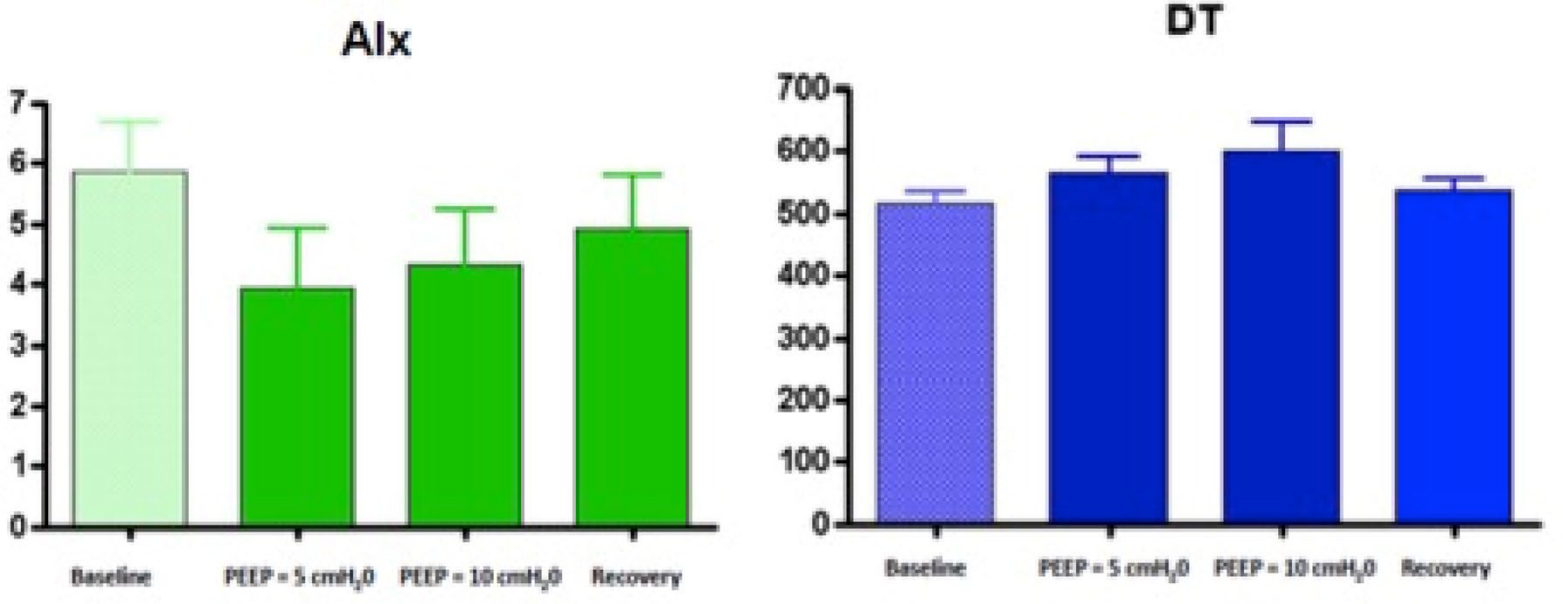
Clustered column for AIx (chart on the left) and DT (chart on the right) from baseline to Recovery; no significant difference observed between the measurements. Data are presented as mean, error bars represent standard deviation. CTR, percentage of changes in comparison to control; AIx, augmentation index; DT, Diastolic time.

Hemodynamic parameters obtained by applanation tonometry are shown in Table 2. There were no significant changes in HR, AIx, LVET, DT through all the different steps: however AIx and DT showed 2 different trends (Fig. 1).

**Table 2:** CWA parameters of the population in study.

Parameters	Baseline	Peep = 5 cmH_2_0	Peep = 10 cmH_2_0	Recovery
AIx (%)	5.85 ± 6.44	3.95 ± 7.56	4.3 ± 27.16	4.92 ± 6.74
Ti (ms)	92.02 ± 24.17	102.4 ± 26.9	104.07 ± 21.52	101.1 ± 23.8
LVET (ms)	297.52 ± 24.32	304.15 ± 35.55	309.47 ± 64.09	300.02 ± 22.84
DT (ms)	516.8 ± 146.8	564.45 ± 203.86	601.13 ± 358.1	536.87 ± 150.63
SEVR	1.41 ± 0.32	1.53 ± 0.38	1.57 ± 0.52	1.47 ± 0.34
PP (mmHg)	40.15 ± 8.83	38.73 ± 8.22	40.27 ± 8.06	39.45 ± 8.91
AP (mmHg)	5.29 ± 3.02	4.84 ± 2.40	5.19 ± 2.95	4.89 ± 2.44
MSBP (mmHg)	105.77 ± 9.99	104.71 ± 10.33	105.27 ± 10.15	105.40 ± 9.82
MDBP (mmHg)	86.53 ± 8.28	86.73 ± 8.83	86.44 ± 8.77	86.58 ± 8.80

Data are expressed as mean ± standard deviation (SD).

PEEP, Positive end-expiratory pressure; AIx, augmentation index; Ti, time to inflection point; LVET, Left Ventricular Ejection Time; DT, Diastolic time; SEVR, diastolic AUC divided by systolic-AUC (Buckberg index); PP, Pulse pressure; AP, Augmentation pressure; MSBP, Mean systolic blood pressure; MDBP, Mean diastolic blood pressure; ms: milliseconds.

**Figure 2:**
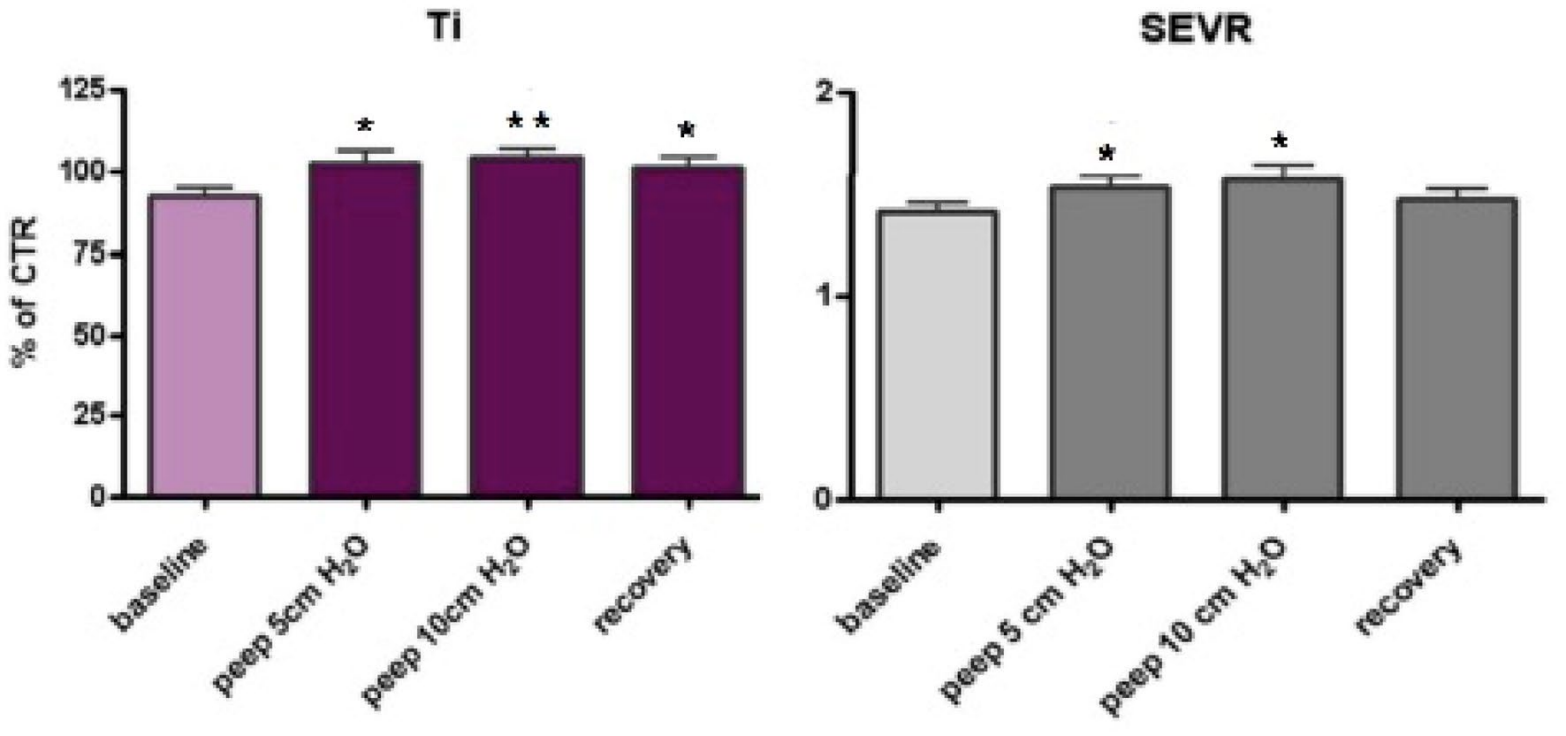
Clustered column for Ti (chart on the left) and SEVR (chart on the right) from baseline to Recovery; significant difference observed for Ti from baseline to PEEP = 10 cmH_2_O and from baseline to recovery; significant increase also for SEVR from baseline to the application of PEEP = 5 cmH_2_O and from baseline to the application of PEEP = 10 cmH_2_O. Data are presented as mean, error bars represent standard deviation. CTR, percentage of changes in comparison to control; Ti, time to inflection point; SVER, diastolic AUC divided by systolic-AUC (Buckberg index). *p values < 0.05, statistically significant. **p values < 0.01.

There was an increase of Ti (ms), significant from baseline to the application of PEEP = 5 cmH_2_O (from 92.02 ± 24.17 to 102.40 ± 26.90; *p* < 0.05), from baseline to PEEP = 10 cmH_2_O (from 92.02 ± 24.17 to 104.07 ± 21.52; *p* < 0.01) and from baseline to spontaneous rebreathing (from 92.02 ± 24.17 to 101.08 ± 23.80; *p* < 0.05) (Fig. 2).

During all the steps of the study there was an increase of SEVR that was significant from baseline to the application of PEEP = 5 cmH_2_O (from 1.41 ± 0.32 to 1.53 ± 0.38; *p* < 0.05) and from baseline to the application of PEEP = 10 cmH_2_O (from 1.41 ± 0.32 to 1.57 ± 0.52; *p* < 0.05) (Fig. 2).

AIx and Ti were significantly correlated at the baseline (*p* < 0.01) and during the application of PEEP = 5 cmH_2_O (*p* < 0.05), while AP and Ti resulted correlated during the application of PEEP = 10 cm H_2_O (*p* < 0.05).

Ti and DT were significantly associated at the baseline and during the application of PEEP = 5 cmH_2_O (both *p* < 0.01).

## Discussion

In our observational study, we evaluated the hemodynamic variations in wave reflection induced by the application of increasing levels of PEEP in healthy subjects. In healthy volunteers, cardiovascular compliance and intravascular status can be assumed to be normal. Consequently, the direct effects of progressive higher levels of PEEP on intact cardiac and arterial systems can be assessed without confounding factors due to comorbidities and therapies. We found a progressive prolongation of Ti and SEVR during each PEEP application. In addition, we observed an inverse correlation between Ti and AIx and a direct correlation between Ti and DT from baseline to PEEP = 5 cm H_2_O. No significant changes were found in heart rate, mean systolic and mean diastolic blood pressure among all stepwise of PEEP.

The cardiovascular response to PEEP depends on several factors; intravascular volume status, cardiac compliance, arterial properties and levels of PEEP. Applanation tonometry provides information on hemodynamic status as well as on arterial wall mechanical properties. The carotid CWA technique allows to obtain surrogate indexes of central hemodynamics and wave reflection, in particular central augmentation index (AIx), timing of the returning reflected waves (Ti) and Buckberg index (SEVR)^12^. These parameters, assessing mechanical arterial properties, are extensively used as an aid to the cardiovascular risk stratification for atherosclerotic-related diseases^18^. AIx depends on age, gender, weight, arterial pressure, LDL concentration and position; several studies shows that AIx independently predicts adverse cardiac events^13,26,27^. Ti, together with reflected wave magnitude, contributes to systolic pressure and is a supposed indicator of aortic stiffness^28^. Buckberg index is a ratio between systolic and diastolic portions of AUCs in the central aortic pulse wave. Blood flow within the coronary arteries occurs mainly during diastole, consequently, the diastolic-AUC indicates myocardial perfusion and oxygen supply. In normal coronary arteries, subendocardial ischemia occurs when SEVR% falls below 50%^29^. Arterial CWA appears to be also a promising non-invasive tool to evaluate arterial properties and ventricular-arterial function in the critical care setting. In our study we observed a transient prolongation of Ti during PEEP ventilation, suggesting a delay of reflected waves and their shift on diastole. This observation can be putatively ascribed to a decrease of arterial peripheral resistance that is directly correlated with diastolic blood pressure and inversely correlated with cardiac output (CO). However, we did not observe significant changes in MDBP during all PEEP steps, and consequently, the arterial peripheral resistance reduction could be the result of an increase of CO. The evaluation of CO is difficult to assess without echocardiographic or invasive measurements but, from a physiological point of view, the possible increase in CO could be in contrast with PEEP effects on preload reduction but in agreement with a decrease in left ventricle afterload ^9^. In addition there was an increase of SEVR during the application of both levels of PEEP. Hence, this result can account for an increase of diastolic AUC or a decrease on systolic AUC. This trend, in addition to a decrease in AIx, may reflect a positive effect of PEEP ventilation on the systolic phase. Having recruited healthy patients, we did not observe significant changes in HR, mean systolic and mean diastolic blood pressure among all stepwise of PEEP.

Garcia et al. evaluated the arterial pressure response (i.e., pulse pressure variation, systolic pressure variation) to the Valsalva manoeuvre (VM) in spontaneous breathing patient^30^. The VM consists in a forced expiratory effort against a closed glottis leading to an increase in intrathoracic pressure, similarly to the PEEP effect. They found that hemodynamic variations induced by the VM predict fluid responsiveness in this group of patients. Contrarily from the above-mentioned study, we did not study the fluid responsiveness to VM and our trial included only healthy subject with normal arterial tone. However, this trial shows the impact of the VM on the haemodynamic system.

There are three main limitations in our study. Firstly, the absence of echocardiographic or invasive measurements of CO that is important to assess PEEP effects on pump function. Secondly, for ethical reasons, we did not investigate changes on CWA induced by levels of PEEP higher than 10 cm H_2_O. Thirdly, we recruited only healthy subjects, thus, our findings might not be applicable to the critical care patients or during mechanical ventilation.

Despite these limitations, this preliminary study may contribute to provide a new protocol for assessing the effects of PEEP atrio-ventricular (A-V) coupling in critical patients. It will be important to integrate echocardiographic assessment and lung ultrasonography examination in order to obtain CO measurement and to diagnostic possible lung injuries. In fact, in patients with ARDS or heart failure, there are many cofactors that influence positive effects of PEEP, together with the seriousness of the disease.

## Conclusion

In conclusion, together with the effects on pulmonary ventilation, we observed an influence of PEEP ventilation on hemodynamic status that is confirmed by morphological changing on contour arterial waveform. It seems that in healthy patients there are not adverse effects induced by PEEP. In healthy subjects, low levels of PEEP seem to have an impact on cardiovascular efficiency thanks to the prolongation of the diastolic phase.

## Data Availability

Data in tables and figures.

## References

[1] Bredenberg CE, Kazui T, Webb WR (1978). Experimental pulmonary edema: The effect of positive end-expiratory pressure on lung water. Ann. Thorac. Surg..

[2] Shapiro BA, Cane RD, Harrison RA (1984). Positive end-expiratory pressure therapy in adults with special reference to acute lung injury: A review of the literature and suggested clinical correlations. Crit. Care Med..

[3] Dieperink W, Jaarsma T, van der Horst IC, Nieuwland W, Vermeulen KM, Rosman H (2007). Boussignac continuous positive airway pressure for the management of acute cardiogenic pulmonary edema: Prospective study with a retrospective control group. BMC Cardiovasc. Disord..

[4] Ranieri VM, Eissa NT, Corbeil C, Chasse M, Braidy J, Matar N (1991). Effects of positive end-expiratory pressure on alveolar recruitment and gas exchange in patients with the adult respiratory distress syndrome. Am. Rev. Respir. Dis..

[5] Faria DA, da Silva EM, Atallah AN, Vital FM (2015). Noninvasive positive pressure ventilation for acute respiratory failure following upper abdominal surgery. Cochrane Database System. Rev..

[6] Acosta P, Santisbon E, Varon J (2007). The use of positive end-expiratory pressure in mechanical ventilation. Crit. Care Clin..

[7] Pinsky MR, Desmet JM, Vincent JL (1992). Effect of positive end-expiratory pressure on right ventricular function in humans. Am. Rev. Respir. Dis..

[8] Qvist J, Pontoppidan H, Wilson RS, Lowenstein E, Laver MB (1975). Hemodynamic responses to mechanical ventilation with PEEP: The effect of hypervolemia. Anesthesiology.

[9] Jardin F, Farcot JC (1982). Influence of positive end-expiratory pressure ventilation on left ventricular function (author's transl). La Nouvelle presse medicale.

[10] Maestroni A, Aliberti S, Amir O, Milani G, Brambilla AM, Piffer F (2009). Acute effects of positive end-expiratory pressure on left ventricle diastolic function in healthy subjects. Int. Emerg. Med..

[11] Luecke T, Pelosi P (2005). Clinical review: Positive end-expiratory pressure and cardiac output. Crit. Care.

[12] Mukkamala R, Xu D (2010). Continuous and less invasive central hemodynamic monitoring by blood pressure waveform analysis. Am. J. Physiol. Heart Circ. Physiol..

[13] Weber T, Auer J, O'Rourke MF, Kvas E, Lassnig E, Lamm G (2005). Increased arterial wave reflections predict severe cardiovascular events in patients undergoing percutaneous coronary interventions. Eur. Heart J..

[14] Lansdorp B, Ouweneel D, de Keijzer A, van der Hoeven JG, Lemson J, Pickkers P (2011). Non-invasive measurement of pulse pressure variation and systolic pressure variation using a finger cuff corresponds with intra-arterial measurement. Br. J. Anaesth..

[15] Desebbe O, Joosten A, Suehiro K, Lahham S, Essiet M, Rinehart J (2016). A novel mobile phone application for pulse pressure variation monitoring based on feature extraction technology: A method comparison study in a simulated environment. Anesth. Analgesia.

[16] Pereira T, Correia C, Cardoso J (2015). Novel methods for pulse wave velocity measurement. J. Med. Biol. Eng..

[17] Drzewiecki GM, Melbin J, Noordergraaf A (1983). Arterial tonometry: Review and analysis. J. Biomech..

[18] Salvi P, Lio G, Labat C, Ricci E, Pannier B, Benetos A (2004). Validation of a new non-invasive portable tonometer for determining arterial pressure wave and pulse wave velocity: The PulsePen device. J. Hypertens..

[19] Boidin MP (1982). A portable PEEP valve for 0–20 cm H2O. Acta Anaesthesiol. Belg..

[20] Kips JG, Schutte AE, Vermeersch SJ, Huisman HW, Van Rooyen JM, Glyn MC (2011). Comparison of central pressure estimates obtained from SphygmoCor, Omron HEM-9000AI and carotid applanation tonometry. J. Hypertens..

[21] Van Bortel LM, Balkestein EJ, van der Heijden-Spek JJ, Vanmolkot FH, Staessen JA, Kragten JA (2001). Non-invasive assessment of local arterial pulse pressure: Comparison of applanation tonometry and echo-tracking. J. Hypertens..

[22] Crilly M, Coch C, Bruce M, Clark H, Williams D (2007). Indices of cardiovascular function derived from peripheral pulse wave analysis using radial applanation tonometry: A measurement repeatability study. Vasc. Med. (London, England).

[23] Charan J, Biswas T (2013). How to calculate sample size for different study designs in medical research?. Indian J. Psychol. Med..

[24] Al-Hashmi KM, Al-Abri MA, Al-Zakwani IS, Al-Waili KA, Al-Rasadi KH, Al-Sabti HA (2014). Reference values for arterial stiffness indices in an Omani Arab population. Saudi Med. J..

[25] Bakker JP, Campbell AJ, Neill AM (2011). Pulse wave analysis in a pilot randomised controlled trial of auto-adjusting and continuous positive airway pressure for obstructive sleep apnoea. Sleep Breath. Schlaf. Atmung..

[26] Weber T, Auer J, O'Rourke MF, Kvas E, Lassnig E, Berent R (2004). Arterial stiffness, wave reflections, and the risk of coronary artery disease. Circulation.

[27] London GM, Blacher J, Pannier B, Guerin AP, Marchais SJ, Safar ME (2001). Arterial wave reflections and survival in end-stage renal failure. Hypertension.

[28] Westerhof BE, Westerhof N (2012). Magnitude and return time of the reflected wave: The effects of large artery stiffness and aortic geometry. J. Hypertens..

[29] Hoffman JI, Buckberg GD (1978). The myocardial supply: Demand ratio—A critical review. Am. J. Cardiol..

[30] Monge Garcia MI, Gil Cano A, Diaz Monrove JC (2009). Arterial pressure changes during the Valsalva maneuver to predict fluid responsiveness in spontaneously breathing patients. Intensive Care Med..

